# Bombali Virus in *Mops condylurus* Bat, Kenya

**DOI:** 10.3201/eid2505.181666

**Published:** 2019-05

**Authors:** Kristian M. Forbes, Paul W. Webala, Anne J. Jääskeläinen, Samir Abdurahman, Joseph Ogola, Moses M. Masika, Ilkka Kivistö, Hussein Alburkat, Ilya Plyusnin, Lev Levanov, Essi M. Korhonen, Eili Huhtamo, Dufton Mwaengo, Teemu Smura, Ali Mirazimi, Omu Anzala, Olli Vapalahti, Tarja Sironen

**Affiliations:** University of Helsinki, Helsinki, Finland (K.M. Forbes, A.J. Jääskeläinen, I. Kivistö, H. Alburkat, I. Plyusnin, L. Levanov, E.M. Korhonen, E. Huhtamo, T. Smura, O. Vapalahti, T. Sironen);; Maasai Mara University, Narok, Kenya (P.W. Webala); Helsinki University Hospital, Helsinki (A.J. Jääskeläinen, O. Vapalahti);; Public Health Agency of Sweden, Stockholm, Sweden (S. Abdurahman, A. Mirazimi);; University of Nairobi, Nairobi, Kenya (J. Ogola, M.M. Masika, D. Mwaengo, O. Anzala);; Karolinska University Hospital, Stockholm (A. Mirazimi);; National Veterinary Institute, Uppsala, Sweden (A. Mirazimi)

**Keywords:** bat, Ebola virus, filovirus, viruses, Kenya, Bombali Ebola virus, *Mops condylurus*, Angolan free-tailed bat

## Abstract

Bombali virus (genus Ebolavirus) was identified in organs and excreta of an Angolan free-tailed bat (*Mops condylurus*) in Kenya. Complete genome analysis revealed 98% nucleotide sequence similarity to the prototype virus from Sierra Leone. No Ebola virus–specific RNA or antibodies were detected from febrile humans in the area who reported contact with bats.

The virus family *Filoviridae* is divided into 5 genera: *Cuevavirus, Marburgvirus, Ebolavirus, Striavirus,* and *Thamnovirus* (https://talk.ictvonline.org/taxonomy). Six distinct Ebola viruses have been described; 4 are known to cause human disease (*1*,*2*). These include highly lethal pathogens capable of producing large outbreaks, namely Bundibugyo, Sudan, and Zaire Ebola viruses, the last responsible for the devastating 2013–2016 outbreak in West Africa and an ongoing extended outbreak in the Democratic Republic of the Congo (*1**,**3**,**4*). Although the natural reservoirs of Ebola viruses remain unconfirmed, considerable evidence supports a role for bat species, particularly fruit bats, analogous to findings implicating *Rousettus aegypticus* fruit bats as a reservoir for Marburg virus (*1**,**5**,**6*).

The most recent Ebola virus to be identified is named Bombali virus (BOMV) and was reported in August 2018 in mouth and fecal swabs collected from free-tailed insectivorous bat species (family Molossidae) *Mops condylurus* and *Chaerephon pumilus* in Sierra Leone (*2*). Although BOMV is not known to infect humans, its envelope glycoprotein shares the same NPC1 receptor as other filoviruses and is capable of mediating BOMV pseudotype virus entry into human cells (*2*). We describe the presence of BOMV in tissues and excreta of an Angolan free-tailed bat (*M. condylurus*) captured near the Taita Hills in southeastern Kenya, the easternmost distributional range of this bat species (*7*), >5,500 km from the original BOMV identification site in Sierra Leone (Figure 1). We also screened human serum samples collected from febrile patients in the Taita Hills area for markers of BOMV infection.

**Figure 1:**
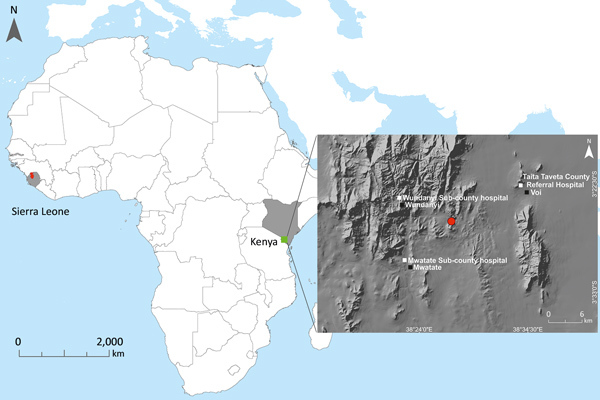
Locations of Bombali Ebola virus infection in Sierra Leone (gray shading at left; Bombali district in red) and Kenya (gray shading at right; Taita Hills area in green). Inset map shows collection site of the Bombali virus–positive bat (red dot) in Kenya, clinics in which human serum samples were collected (white squares), and the closest towns (black squares).

We identified BOMV in an adult female bat (B241) by reverse transcription PCR and next-generation sequencing. This bat was captured along with 15 others in mist nets in savannah habitat near a small river in May 2018; only this bat was BOMV positive; (6% prevalence). Viral RNA was present in lung, spleen, liver, heart, intestine, mouth swab, and fecal samples but absent from the brain, kidney, urine, and a few fleas found on the bat; viral loads were especially high in the lung (Appendix). These tissue-positive findings confirm that BOMV can infect *M. condylurus* and is not an artifact of its insect diet, which could not be discounted from the previous analysis on the basis of mouth and fecal swabs (*2*). We also screened lung samples of sympatric *C. pumilus* bats (n = 13) and other bat species (Appendix Table 2) captured from the same area in February 2016 and May 2018; all were negative for BOMV RNA. Serologic analysis revealed antibodies against Ebola virus in the blood of the tissue-positive bat (Appendix Figure), but specific antibodies were not found in blood from the other bats (Appendix).

Our tissue-positive findings provide a strong host association between BOMV and *M. condylurus *bats; it is possible that BOMV–positive findings from other bat species result from local spillover or contamination. Moreover, phylogenetic analysis of the full BOMV genome from the bat lung revealed 98% nucleotide sequence similarity with the prototype reported in Sierra Leone (GenBank accession no. MK340750) (Figure 2). Considering the high sequence similarity between the 2 locations and that *M. condylurus* bats, like most insectivorous bats, are believed to travel only short distances (*8*), BOMV is likely to be distributed throughout much of sub-Saharan Africa (*7*). However, further monitoring of *M. condylurus* and *C. pumilus* bats and other sympatric species across Africa is required to support this hypothesis.

**Figure 2:**
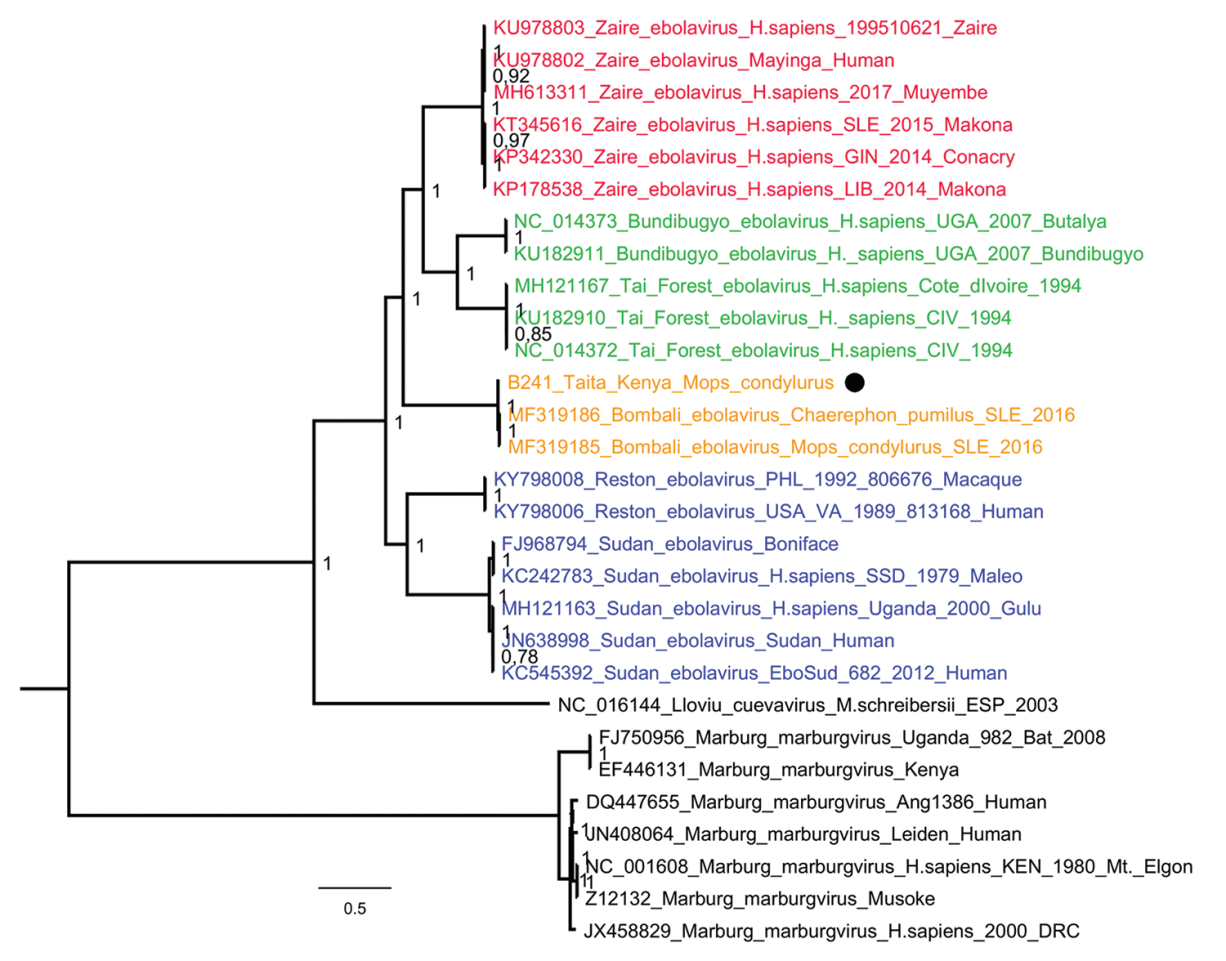
Phylogenetic tree of complete filovirus genomes (18,795–19,115 nt), including Bombali Ebola virus in Sierra Leone and now Kenya (19,026 nt; black dot). Representative sequences were retrieved from the Virus Pathogen Database and Analysis Resource and aligned with a MAFFT online server (http://mafft.cbrc.jp/alignment/software). The tree was built using the Bayesian Markov Chain Monte Carlo method, using a general time reversible model of substitution with gamma-distributed rate variation among sites allowing the presence of invariable sites. Posterior probabilities are shown at the nodes. Scale bar indicates genetic distance.

Because *M. condylurus* bats commonly roost in human structures, such as house roofs (*8**,**9*), human exposure to this species is more likely than for many other bat species. Therefore, we screened for markers of human infection with BOMV by studying serum samples collected from febrile patients who sought treatment at clinics in the Taita Hills area during April–August 2016. Clinics are located in the surrounding areas, all within 15 km of the BOMV–infected bat collection site (Figure 1). We screened patients for filovirus RNA (n = 81) and Ebola virus–specific IgG (n = 250) by an immunofluorescence assay using Zaire Ebola virus VP40–transfected VeroE6 cells as antigen (Appendix). Many samples, including all those screened for filovirus RNA, were from patients who reported contact with bats in the home or workplace. We found no evidence of filovirus infection by either screening method, providing no support that BOMV easily infects humans or is a common cause of febrile illness in the area. Ongoing surveillance is nonetheless necessary, and we cannot exclude the possibility that BOMV was a recent introduction to the Taita Hills area.

Our results markedly expand the distributional range of this new Ebola virus to eastern Africa and confirm the *M. condylurus* bat as a competent host. Like Goldstein et al. (*2*), we stress that the virus is not known to infect humans, a premise supported by our screening of febrile patients in the Taita Hills area. Potential efforts to eradicate bats are unwarranted and may jeopardize their crucial ecosystem roles and human health (*10**,**11*).

Appendix Additional information about Bombali Ebola virus in *Mops condylurus* bat, Kenya.
