# Supplementary material for: Bombali Virus in Mops condylurus Bat, Kenya
Source: Emerg Infect Dis. 2019 May;25(5):955–7. doi: 10.3201/eid2505.181666 (PMC6478230; doi:10.3201/eid2505.181666)
Supplement: Appendix — Additional information about Bombali Ebola virus in Mops condylurus bat, Kenya. [file 18-1666-Techapp-s1.pdf]

Article DOI: https://doi.org/10.3201/eid2505.181666

# Bombali Ebola Virus in *Mops condylurus* Bat, Kenya

## Appendix

## Methods

Bats were captured in February 2016 and May 2018 as part of an ongoing virus screening project in the Taita Hills area of rural Kenya; all *M. condylurus* were captured in 2018. We employed mist and hand netting, and structured trapping site selection to focus on habitat and species diversity and minimize the number of individuals collected from any 1 species or site. Captured bats were placed into individual cotton bags, and processed at the University of Helsinki Taita Research Station. Species identifications were made in the field using keys (*1*). Non-conservation priority bat species (classified as least concern by the IUCN) were euthanized via cervical dislocation to collect blood, lung, liver, spleen, kidney, intestine and brain samples, as well as urine, feces, and ectoparasites when possible. Dissections were performed in a sheltered outside area, using personal protective equipment, including FFP3 facemasks, latex gloves, and safety gowns. Bat tissues were placed into separate marked tubes with RNAlater (Sigma, https://www.sigmaaldrich.com), stored at −20°C, and later sent on dry ice to Helsinki, Finland.

At the University of Helsinki, under enhanced BSL-3 conditions, bat tissue samples were treated with Tripure (Roche, http://www.roche.com) to inactivate any potential hazardous agents before RNA extractions (Tripure method) and screening by a pan-filovirus RT-qPCR (*2*). Filovirus screening was initially conducted as a precaution, to facilitate screening for other viruses under less-strict biosafety conditions. The pan-filo RT-qPCR has been tested to detect Zaire EBOV, Bundibugyo, Sudan, Taï Forest, and Reston ebolavirus, in addition to Marburg virus (MARV) and Ravn Virus RNA. EBOV and MARV RNA were used as positive controls (in vitro RNAs) (*3*). Following the identification of a positive individual, with particularly high viral loads in the lung, lung samples from all bats were also screened with a Bombali virus–specific

real-time RT-PCR (*4*). All tissue, excreta, and ectoparasite samples were screened from the positive individual (Appendix Table 1), and viral loads determined by RT-qPCR with an in vitro transcribed RNA serving as the quantification standard. A full list of each bat species captured and screened is provided in Appendix Table 2.

Prior to whole-genome sequencing, RT-PCR positive samples were treated with DNase I (Thermo Fisher, http://www.thermofisher.com), and purified with Agencourt RNA Clean XP magnetic beads (Beckman Life Sciences, https://www.beckman.com). Ribosomal RNA was removed using a NEBNext rRNA depletion kit (New England BioLabs, https://www.neb.com), according to the manufacturer’s protocol. The sequencing library was prepared using a NEBNext Ultra II RNA library prep kit (New England BioLabs). Libraries were quantified using a NEBNext Library Quant kit for Illumina (New England BioLabs). Pooled libraries were then sequenced on a MiSeq platform (Illumina, https://www.illumina.com) using a MiSeq v3 reagent kit with 300 bp paired-end reads. Raw sequence reads were trimmed and low-quality (quality score <15) and short (<36 nt) sequences were removed using Trimmomatic (*5*). Thereafter, de novo assembly was conducted using MegaHit (*6*). Open reading frames were sought using MetaGeneAnnotator (*7*), followed by taxonomic annotation using SANSparallel (*8*). We confirmed bat species identity of the positive individual by retrieving cytochrome-b sequences from the NGS reads (GenBank accession no. MK330941).

The phylogenetic tree was constructed using the Bayesian Markov chain Monte Carlo (MCMC) method, implemented in Mr Bayes version 3.2 (*9*) using a GTR-G-I model of substitution with 2 independent runs and 4 chains per run. The analysis was run for 5 million states and sampled every 5,000 steps. The average standard deviation of split frequencies was 0.000732.

Febrile patients seeking care at 3 health facilities in the Taita Hills (Wundanyi, Mwatate, and Voi) were recruited into the study by clinicians. A questionnaire was used to capture socio-demographic data and pertinent history, including a tickbox question regarding contact with bats at home or work. Based on the criterion of exposure to bats, a total of 81 patients (2.9–83.4 years of age; average, 38.8 years) were selected for analysis of filovirus RNA. Samples were collected within 5 days of the onset of fever. No patients reported bleeding. Reported symptoms included, in addition to fever; myalgia (54/81), joint pain (45/81), rash (9/81), diarrhea (8/81), vomiting

(7/81), headache (6/81) and cough (4/81). Serum samples were stored at the University of Helsinki Taita Research station at −20°C for ≤3 weeks, and then transported on ice to a central laboratory at the University of Nairobi where they were stored at −80°C and later shipped on dry ice to Helsinki. Nucleic acids were extracted from 100µL of serum and eluted to 50µL using the QIAamp Viral RNA Mini Kit (QIAGEN, https://www.qiagen.com) according to manufacturer's instructions. Pan-filovirus RT-qPCR was then conducted as described above, as well as Bombali virus–specific RT-PCR (*4*).

Human serum samples were analyzed for Ebola virus–specific IgG antibodies using an immunofluorescence assay (IFA) based on a recombinant Zaire ebolavirus VP-40 with a similar IFA protocol as described before (*10*), and demonstrated within the EbolaMoDRAED EU-IMI project to react with Zaire ebolavirus patient serum. Bombali virus VP40 protein is 75%–78% similar to that of other ebolaviruses, which have been demonstrated to cross-react within the genus (*11*). As antigen, we used acetone-fixed Vero E6 cells transfected with the pCAGGS-Ebola VP40 construct (Zaire ebolavirus, isolate Ebola virus/ H.sapiens-wt/SLE/2014/Makona-G3856.1 sequence, GenBank KM233113.1), and as controls, cells transfected with the empty vector. Patient serum samples were diluted 1:60 in PBS and incubated for 1 h at 37°C. Fluorescein isothiocyanate–conjugated anti-human IgG (Jackson ImmunoResearch, https://www.jacksonimmuno.com) was diluted 1:30 in PBS, and incubated for 30 min at 37°C. Unbound antibodies and anti-human IgG were washed 3 times with PBS and then once with distilled water. The slides were covered with mounting medium and coverslips, and read using a ×20 objective of fluorescence microscope Olympus IX71 (Olympus Corporation, www.olympus-global.com).

## Additional Results

Serologic analysis revealed antibodies against ebolavirus in the blood of the tissue-positive bat (Appendix Figure), but antibodies were not present in blood from the other bats. Note that bat blood samples (from RNA-negative individuals) were first heat inactivated under enhanced BSL-3 conditions. To minimize exposure risk, the blood sample from the positive bat was sent to the Public Health Agency of Sweden and screened under BSL-4 conditions. To detect bat antibodies in blood samples, Vero E6 cells transfected as above to produce ZEBOV

VP40, or at Public Health Agency of Sweden, infected with Zaire ebolavirus, were used in IFA according to a previously described protocol (*12*). Blood samples were diluted to 1:20 in PBS before incubation. Detection was done with goat anti–bat antibody Ig (Bethyl Laboratories, https://www.bethyl.com) at 1:1,000, followed by donkey anti–goat cyanin 2 (Cy2)-labeled Ig (Dianova, https://www.dianova.com) at 1:100. Slide staining and analysis were conducted as described above.

## References


1. Patterson BD, Webala PW. Keys to the bats (Mammalia: *Chiroptera*) of East Africa. Fieldiana Life Earth Sci. 2012;6:1–60. http://dx.doi.org/10.3158/2158-5520-12.6.1

2. Jääskeläinen AJ, Sironen T, Tidiane Diagne C, Moïse Diagne M, Faye M, Faye O, et al. Development, validation, and clinical evaluation of a broad-range pan-filovirus RT-qPCR applicable in freeze-dried format. J Clin Viol. In press 2019.

3. Jääskeläinen AJ, Moilanen K, Aaltonen K, Putkuri N, Sironen T, Kallio-Kokko H, et al. Development and evaluation of a real-time EBOV-L-RT-qPCR for detection of *Zaire ebolavirus*. J Clin Virol. 2015;67:56–8. PubMed http://dx.doi.org/10.1016/j.jcv.2015.04.003

4. Goldstein T, Anthony SJ, Gbakima A, Bird BH, Bangura J, Tremeau-Bravard A, et al. The discovery of Bombali virus adds further support for bats as hosts of ebolaviruses. Nat Microbiol. 2018;3:1084–9. PubMed http://dx.doi.org/10.1038/s41564-018-0227-2

5. Bolger AM, Lohse M, Usadel B. Trimmomatic: a flexible trimmer for Illumina sequence data. Bioinformatics. 2014;30:2114–20. PubMed http://dx.doi.org/10.1093/bioinformatics/btu170

6. Li D, Liu CM, Luo R, Sadakane K, Lam TW. MEGAHIT: an ultra-fast single-node solution for large and complex metagenomics assembly via succinct de Bruijn graph. Bioinformatics. 2015;31:1674–6. PubMed http://dx.doi.org/10.1093/bioinformatics/btv033

7. Noguchi H, Taniguchi T, Itoh T. MetaGeneAnnotator: detecting species-specific patterns of ribosomal binding site for precise gene prediction in anonymous prokaryotic and phage genomes. DNA Res. 2008;15:387–96. PubMed http://dx.doi.org/10.1093/dnares/dsn027

8. Somervuo P, Holm L. SANSparallel: interactive homology search against Uniprot. Nucleic Acids Res. 2015;43(W1):W24-W29. PubMed http://dx.doi.org/10.1093/nar/gkv317

9. Ronquist F, Teslenko M, van der Mark P, Ayres DL, Darling A, Höhna S, et al. MrBayes 3.2: efficient Bayesian phylogenetic inference and model choice across a large model space. Syst Biol. 2012;61:539–42. PubMed http://dx.doi.org/10.1093/sysbio/sys029

10. Levanov L, Kuivanen S, Matveev A, Swaminathan S, Jääskeläinen-Hakala A, Vapalahti O. Diagnostic potential and antigenic properties of recombinant tick-borne encephalitis virus subviral particles expressed in mammalian cells from Semliki Forest virus replicons. J Clin Microbiol. 2014;52:814–22. PubMed http://dx.doi.org/10.1128/JCM.02488-13

11. Natesan M, Jensen SM, Keasey SL, Kamata T, Kuehne AI, Stonier SW, et al. Human survivors of disease outbreaks caused by Ebola or Marburg virus exhibit cross-reactive and long-lived antibody responses. Clin Vaccine Immunol. 2016;23:717–24. PubMed http://dx.doi.org/10.1128/CVI.00107-16

12. Binger T, Annan A, Drexler JF, Müller MA, Kallies R, Adankwah E, et al. A novel rhabdovirus isolated from the straw-colored fruit bat *Eidolon helvum*, with signs of antibodies in swine and humans. J Virol. 2015;89:4588–97. PubMed http://dx.doi.org/10.1128/JVI.02932-14

**Appendix Table 1.** Viral loads from *Mops condylurus* bat that tested positive for Bombali Ebola virus.

| Sample | Ct value | Copy number/500 ng total RNA |
|---|---|---|
| Mouth swab† | 24.00 | Not applicable |
| Spleen | 32.76 | 414 |
| Liver | 33.95 | 181 |
| Intestine | 32.76 | 413 |
| Heart | 29.82 | 3,173 |
| Feces | 29.14 | 5,121 |
| Lung | 16.74 | 27,950,000 |
| Kidney | Negative | 0 |
| Urine | Negative | 0 |
| Fleas | Negative | 0 |

*Viral loads for each sample type were estimated using a standard curve based on in vitro transcribed and quantified RNA.
†Mouth swab has no copy number because it was screened in a BSL-4 laboratory in Sweden using a different protocol and without the standard curve.

**Appendix Table 2.** Bat species screened for filoviruses, Kenya*

| Species | 2016 | 2018 |
|---|---|---|
| *Mops condylurus* | 0 | 16 |
| *Chaerephon pumilus* | 4 | 7 |
| *Cardioderma cor* | 36 | 20 |
| *Chaerephon chapini* | 1 | 0 |
| *Epomophorus wahlbergi* | 19 | 23 |
| *Glauconycteris argentata* | 1 | 0 |
| *Hipposideros caffer* | 2 | 1 |
| *Lavia frons* | 0 | 1 |
| *Lissonycteris angolensis* | 10 | 0 |
| *Myotis tricolor* | 1 | 0 |
| *Neoromicia nana* | 0 | 3 |
| *Nycticeinops schlieffeni* | 3 | 1 |
| *Nycteris thebaica* | 0 | 1 |
| *Rhinolophus clivosus* | 0 | 2 |
| *Rhinolophus landeri* | 1 | 2 |

| Species | 2016 | 2018 |
|---|---|---|
| *Rousettus aegyptiacus* | 17 | 13 |
| *Scotoecus hirundo* | 34 | 4 |
| *Scotophilus dinganii* | 4 | 12 |
| *Rousettus lanosus* | 0 | 1 |
| *Pipistrellus sp.* | 20 | 0 |
| *Neoromicia sp.* | 0 | 1 |
| *Miniopterus sp.* | 6 | 0 |
| *Hypsugo sp.* | 4 | 0 |

*Bats were captured from the Taita Hills area in 2016 and 2018. All bat lung samples were screened for filovirus RNA via a new pan-filovirus reverse transcription qualitative PCR (*2*) and a Bombali virus–specific real-time reverse transcription PCR (*4*).

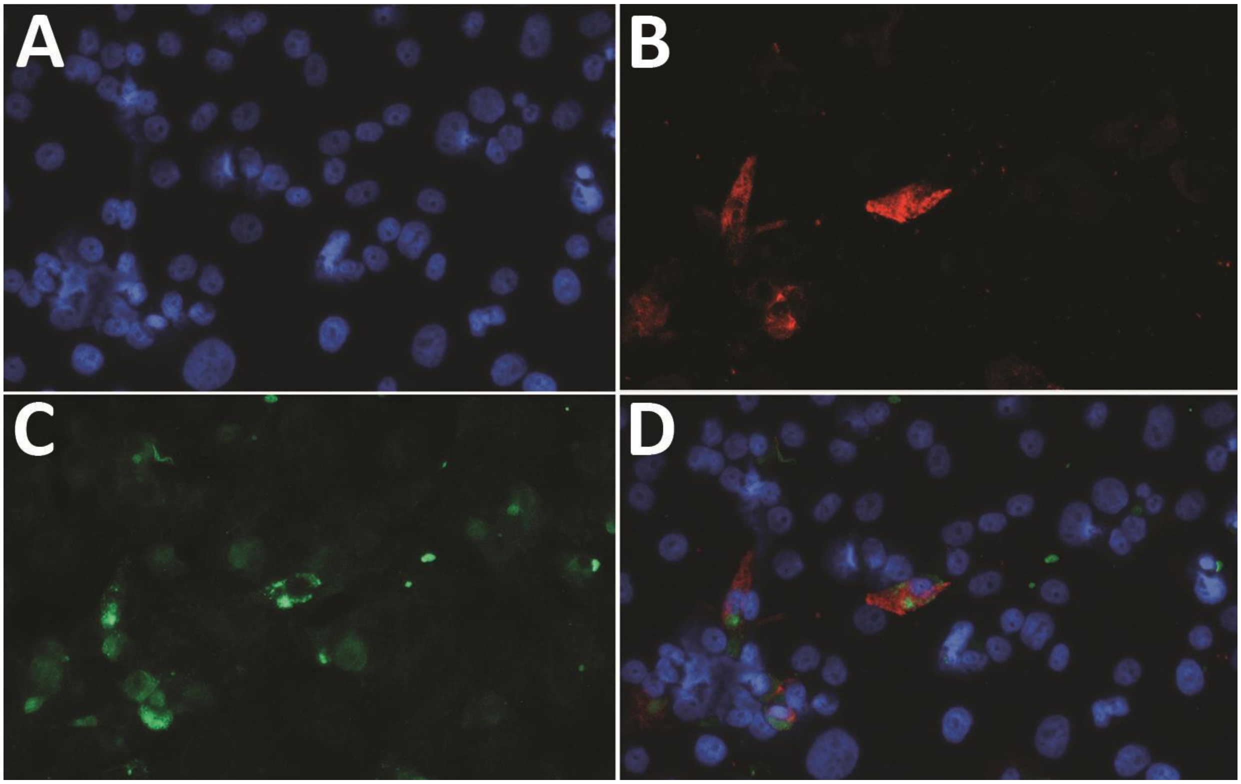


**Appendix Figure**. Detection of Ebola virus–specific antibodies in Bat B241 (the BOMV RNA positive individual) using an immunofluorescence assay based on Zaire ebolavirus (ZEBOV)–infected, acetone-fixed Vero E6 cells. The slides contain ZEBOV-infected and noninfected control cells. A) 4′,6-diamidino-2-phenylindole (DAPI) staining for cell nuclei. B) Staining with rabbit anti–ZEBOV-GP showing ZEBOV-infected cells. C) Staining with bat B241 serum at a dilution of ≈1:200, demonstrating specific granular staining of ZEBOV-infected cells. D) A merge of stains demonstrating that the antibody response of bat B241 is Ebola virus genus cross-reactive, but targeting other viral proteins than the ZEBOV GP.
